# Measuring fatigue four years after stroke: a bi-factor analysis of the Norwegian Chalder Fatigue Questionnaire

**DOI:** 10.1186/s41687-026-01151-9

**Published:** 2026-07-21

**Authors:** Synne G. Pedersen, Oddgeir Friborg, Audny Anke, Marianne B. Halvorsen, Mari T. Løkholm, Marte Christine Ørbo

**Affiliations:** 1 Department of Rehabilitation, University Hospital of North Norway, Tromsø, Norway; 2 Institute of Health and Society, Research Centre for Habilitation and Rehabilitation Model and Services (CHARM), Faculty of Medicine, University of Oslo, Oslo, Norway; 3 Department of Clinical Medicine, Faculty of Health Sciences, UiT - The Arctic University of Norway, Tromsø, Norway; 4 Department of Psychology, Faculty of Health Sciences, UiT - The Arctic University of Norway, Tromsø, Norway; 5 Vigor Rehabilitation Hospital, Tromsø, Norway

**Keywords:** The Chalder Fatigue Questionnaire (CFQ), Dimensionality, Post-stroke fatigue, Stroke

## Abstract

**Background:**

Post-stroke fatigue (PSF) is a persistent and debilitating condition. Assessing its characteristics and severity accurately is important, but the suitability of existing fatigue instruments for use in stroke populations is uncertain. The Chalder Fatigue Questionnaire (CFQ) is a widely used generic measure capturing both mental and physical fatigue, but the dimensionality of the 11-item CFQ is debated. The objective of this study was to examine the best-fitting model for the CFQ and evaluate whether the addition of latent specific factors for the physical and mental items provided meaningful information beyond the latent general fatigue score.

**Methods:**

We examined three competing measurement model of the CFQ – unidimensional, correlated two-factor (physical and mental fatigue) and a bi-factor model in a sample of 144 individuals at 4 years post-stroke. Furthermore, we investigated how other criterion-related variables correlated with the estimated CFQ factor scores compared with the simple CFQ.

**Results:**

The bi-factor measurement model had best fit. Partial correlation analyses indicated that a simple overall fatigue score aligns relatively well with the estimated general factor score, as does the physical sum score. This indicates that separate physical and mental sum scores provide no additional value, whereas their estimated latent specific factors contribute more information. Men reported less fatigue, and except for gender most of the demographic variables were not significantly correlated with the CFQ variables.

**Conclusion:**

Despite best psychometric support for a bi-factor measurement model, a simple overall sum score may be used to assess fatigue severity satisfactorily. While separate physical and mental factors provide noteworthy nuance, their contribution is in practice limited. Future research should consider refining the mental fatigue items if a more precise assessment or a more nuanced clinical applicability of the physical-mental separation following stroke is needed.

## Abstract (plain language summary)

Many people feel tired after having a stroke. This is called post-stroke fatigue. It can make daily life difficult, so doctors and researchers need to measure it accurately to offer the right support. The researchers looked at a commonly used questionnaire called the Chalder Fatigue Questionnaire (CFQ). It asks about both physical tiredness (like feeling weak or heavy) and mental tiredness (like difficulty concentrating). The study explored whether this questionnaire works well for people who have had a stroke, and whether it’s helpful to separate fatigue into physical and mental types. The study included 144 people who had a stroke four years ago. It showed that a simple overall score from the questionnaire gave a clear and accurate picture of a person’s fatigue. While the physical fatigue questions gave useful information, the mental fatigue questions did not add much extra. The study found that men reported less fatigue than women, but other participants characteristics did not make much difference. In conclusion, the CFQ is suitable for measuring overall fatigue after stroke, but further work is needed to improve how mental fatigue is assessed. This could lead to better support for people experiencing post-stroke fatigue.

## Introduction

Post-stroke fatigue (PSF) is one of the most common and debilitating consequences of stroke, often persisting long after the initial event [1, 2] and is commonly defined as a long-term subjective feeling of lack of energy, weariness, and aversion to effort that interferes with daily activities [3]. Long-term or persisting fatigue is associated with reduced health-related quality of life [4, 5], impaired functioning [2, 6], and increased risk of mental health issues such as depression and anxiety [7, 8]. The combination of high prevalence in which fatigue affect every other stroke patient [9], and the substantial health-related negative impact, makes fatigue an important target for treatment and prevention. However, the underlying mechanisms of PSF remains elusive, and the clinical assessment of fatigue may lack the precision to inform treatment or prevention strategies [10].

PSF shares some common underlying mechanisms with other conditions, such as multiple sclerosis (MS), and chronic fatigue syndrome (CFS) [11, 12]. However, the stroke population has unique characteristics that may require specialized assessment tools or adaptations of existing instruments. Generic measures, such as those used in the general population or for other disease groups, may overlook or oversimplify the specific functional impairments and lifestyle disruptions that occur after a stroke. Therefore, it is crucial to evaluate whether these generic measures are sufficiently sensitive to capture stroke-related difficulties. The most used tool for assessing fatigue after stroke is the Fatigue Severity Scale (FSS). However, models of PSF suggest that it has both physical and mental dimensions, with psychological factors becoming more prominent over time as explanatory variables [10]. This highlights the importance of utilizing assessment tools that can distinguish between physical and mental fatigue when measuring PSF several years after a stroke. The Chalder Fatigue Questionnaire provides a nuanced evaluation by separately measuring these two dimensions and has been translated into Norwegian. However, previous research has to a very limited extent investigated if this is a solid instrument in measuring fatigue after stroke [13].

The Chalder Fatigue Questionnaire (CFQ) [14] is a widely used self-report questionnaire for assessing the severity of fatigue in various populations, including stroke [15]. The CFQ consists of 7 and 4 items that assess physical and mental aspects of fatigue, respectively. The physical items include questions about energy levels, and tiredness, whereas the mental item include questions such as difficulty with concentration. There is an ongoing debate regarding the dimensionality of the 11-item CFQ, and we have not found any studies that have examined it among stroke survivors. The CFQ has been subjected to factor analysis in general populations [12, 16, 17], and somatic populations, e.g. Chronic fatigue syndrome [12] and Multiple Sclerosis [11], which have revealed different factor structures. Most studies suggest that the CFQ is best represented as a unidimensional construct [18], whereas others suggest that CFQ reflects multiple dimensions separating mental from physical fatigue [12, 14] or even consists of more than two factors by splitting the physical factor in further two factors [17]. One potential reason for the disparate findings may be the reliance on exploratory factor analysis (EFA), which is entirely data-driven and therefore more prone to extract minor factors associated with sample peculiarities that are not present in other samples. The dimensionality of the CFQ should therefore be examined with confirmatory factor analysis (CFA) methods that are less prone to structural model changes despite variations in minor factors between samples, such as the bi-factor analysis approach [19].

A known limitation of conventional CFA is the highly restrictive requirements that all items correlate with a single factor. This generally works well for multi-dimensional scales with relatively non-overlapping items and few measurements artifacts [20]. However, in scales that purportedly measure multiple but correlated dimensions, items may be associated with more than one source of influence (i.e., additional factors). Such scales, as the CFQ is an example of, will typically need to include extra parameters for unexplained (residual) item correlations [21], item cross-loadings (i.e., unintended item-factor correlations), or artificially crank up the factor correlations to achieve better model fit [22]. Such modelling variations aimed at improving model fit are generally insubstantial and tend to appear or disappear across studies. A better factor analytic question is, therefore, whether the model demonstrates sufficient practical unidimensionality despite some extra specific factors being present. The bi-factor analytic approach is well suited to examine this possibility [23] and is useful for understanding constructs with a broad overarching dimension supplemented by narrower subdimensions. It improves factor interpretation through a clearer distinction between these variance dimensions and prevents factor correlation inflations, imprecision related to item cross-loadings, and poor model fit. This distinction is clinically important. If CFQ is best represented as unidimensional, an overall fatigue score may be sufficient for clinical assessment. However, if physical and mental fatigue factors contribute additional unique information, the separate sub-scores may enhance the precision of fatigue evaluation after stroke.

The overarching objective of this study is to examine which measurement model best represents the CFQ instrument in fatigue assessment in people who previously have suffered a stroke. To this end, we examined the unidimensional model and compared it with the correlated two-factor model, and a bi-factor model to evaluate whether distinguishing between physical and mental fatigue provides meaningful additional information. Given the uneven number of physical and mental fatigue items, and the high correlation between them, we expect the CFQ to reflect a single underlying factor and, therefore, to be unidimensional. Finally, we examined how other criterion-related variables correlate with the general and specific CFQ factors to better understand their potential meaning and clarify their clinical and conceptual significance.

## Methods

This study is part of an observational prospective study, the ‘Fatigue after Stroke study’, with fatigue data collected four years post-stroke. The study is registered in ClinicalTrials.gov (ID: NCT03639259).

### Participants

Participants were recruited from the Norwegian branch of the study ‘Rehabilitation, Function, and Quality of Life after Stroke in North Norway and Central Denmark – the NorDenStroke Study’. Patients with confirmed cerebral stroke were enrolled from one of three stroke units at the University Hospital of North Norway (UNN HF) between March 2014 and December 2015 [24]. Exclusion criteria included strokes associated with brain malignancy, subarachnoid hemorrhage, or brain trauma.

Eligible stroke survivors met the inclusion criteria of the National Norwegian Stroke Register. This was defined clinically based on the World Health Organization’s definition of acute ischemic or hemorrhagic stroke (ICD-10 codes I.63 and I.61, respectively) in individuals aged 18 years or older. For this study, only stroke survivors who had completed questionnaires in the NorDenStroke study one-year post-stroke were eligible (*n* = 217). A dropout analysis was conducted, comparing the 149 participants who completed the fatigue follow-up study four years post-stroke to the 68 stroke survivors who either declined participation or did not respond. Non-responders (*n* = 68) were significantly older (*p* = 0.001) than participants who completed the study (mean age = 72 years, SD = 10.7, vs. mean age = 67 years, SD = 11.0). However, the two groups did not differ in terms of gender, stroke type, or stroke severity. Five participants were excluded from the study due to declining health, which prevented them from completing the questionnaires. Consequently, the total number of participants with complete data included in the present study were 144.

**Fig. 1:**
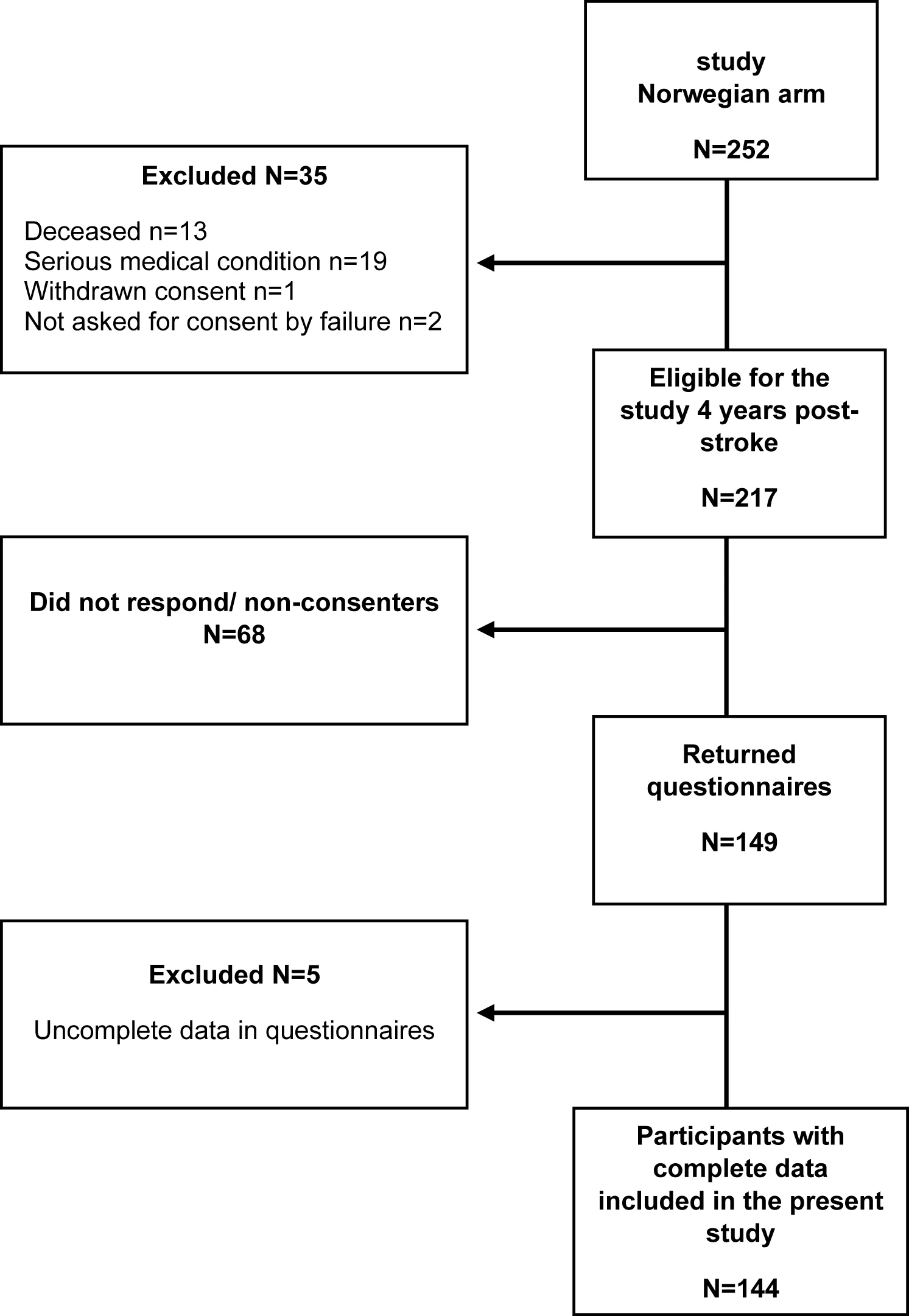
Flowchart

A flowchart adhering to the Strengthening the Reporting of OBservational studies in Epidemiology (STROBE) criteria is presented in Fig. 1.

### Data collection procedures at four-year

Eligible participants were informed about the study by a healthcare professional and asked to provide written consent. Once new written consent was obtained, questionnaires were mailed to participants. In cases of incomplete responses, participants received follow-up phone calls and were encouraged to complete any missing items.

## Measurements

### Primary outcome at four-year follow-up

*The Chalder Fatigue Questionnaire (CFQ)* is a widely used self-report questionnaire designed to assess fatigue in clinical and non-clinical populations. It was first introduced by Chalder et al. in 1993 [14] and has been considered valuable for measuring the severity of fatigue across both physical and mental domains. Physical fatigue (7 items) assesses somatic symptoms, such as feelings of tiredness, muscle weakness, and the need for rest. Mental fatigue (4 items) evaluates cognitive aspects of fatigue, including difficulty concentrating and memory problems. Respondents rate their fatigue severity based on their experiences in the last month using a 4-point Likert scale (0-less than usual, 1-no more than usual, 2-more than usual, and 3-much more than usual). The total score is the sum of each item with a range from 0 to 33.

The present study used the translated Norwegian version which uses a marginally revised version [25], that is, the item “Do you have problems thinking clearly?” was replaced with “Do you find it more difficult to find the correct word?”. The developers state that either item can be used without altering the meaning of the scale [25], but this revision has shown slightly better test score reliability than the original scale [12, 14]. The CFQ has demonstrated high internal consistency in various studies. For instance, a study by Cella & Chalder [12] reported Cronbach’s alpha values ranging from 0.88 to 0.92, indicating strong reliability. Other research has supported satisfactory internal consistency, with omega coefficients ranging from 0.78 to 0.96 [16]. The responsiveness of CFQ have been documented in studies tracking sensitivity to change over time. For example, a study assessing fatigue in patients with multiple sclerosis [11] found that the CFQ was responsive to changes across both low and high-intensity behavioral interventions, demonstrating its utility in monitoring fatigue severity over time.

### Variables at four-year follow-up

*The modified Rankin Scale (mRS)* [26] is a six-item global outcome scale widely used in stroke research to assess patients’ functional abilities and independence in daily activities [27]. It has been a key primary or secondary outcome measure in many recent large-scale stroke trials [28]. The mRS is a validated, clinician-reported tool for evaluating global disability. However, studies have demonstrated its adaptability for self-reported use. One study found strong validity of mRS scores reported by patients six months post-stroke compared to clinician-assessed scores [29]. Similarly, another study highlighted the reliability and efficiency of patient or proxy survey responses in generating mRS scores after subarachnoid hemorrhage, aligning well with results from trained personnel [30]. In this study, we provided participants with detailed descriptions for each functional category in the mRS to facilitate accurate self-assessment.

*The Fatigue Severity Scale (FSS)* [31, 32] assesses patients’ global fatigue levels. Widely employed in population-based stroke research [1], the FSS has demonstrated excellent internal consistency (Cronbach’s alpha = 0.92) and good test-retest reliability (intraclass correlation coefficient = 0.742) [27]. The FSS consists of nine items that evaluate fatigue in daily life across domains such as daily activities, social participation, motivation, and sleep. Each item is rated on a seven-point Likert scale, ranging from 1 (no problem) to 7 (a significant problem). In this study, the Cronbach’s alpha for the nine items ranged from 0.95 to 0.96. A global average score is calculated based on the responses, with higher scores reflecting greater fatigue [32]. Severe fatigue is typically defined as an FSS score of ≥5 [32].

*The Hospital Anxiety and Depression Scale (HADS)* is a widely used tool for assessing non-vegetative symptoms of anxiety and depression, each measured with seven items [33]. Individual item scores range from 0 to 3, resulting in subscale scores ranging from 0 to 21, with higher scores indicating greater symptom severity. A review of 747 studies confirmed the HADS as a valid measure for assessing symptom severity and identifying clinical caseness [34]. Clinically, anxiety or depression is often defined as a HADS score of ≥ 8 [35]. The scale has been extensively used in large Norwegian epidemiological studies [35, 36] and among patients suffering a stroke [37]. In this study, the HADS demonstrated strong internal consistency, with Cronbach’s alpha values of 0.88 for anxiety and 0.81 for depression.

*The Stroke-Specific Quality of Life (SS-QOL) scale* [38] is a multidimensional, stroke-specific health-related quality of life (HRQOL) instrument designed to assess the functional impact of stroke [6, 39, 40]. It comprises 49 items across 12 domains: mobility, energy, upper extremity function, work and productivity, mood, self-care, social roles, family roles, vision, language, thinking, and personality. Each domain is evaluated using three to six items on a 5-point Likert scale, where higher scores indicate better function. Average index scores for each domain allow for comparisons and help identify specific capacities affected by stroke [38]. Previous research has used principal component analysis to explore components within the SS-QOL scale [6, 24, 41], and this study uses the two components ‘physical health’ (PH) and ‘Cognitive-Social-Mental health’ (CSM) as described by Pedersen et al. [6]. The reliability of the SS-QOL domains and overall scores has been well-documented, with studies confirming acceptable internal consistency [38, 39, 42]. Additionally, the test-retest reliability coefficient has been shown to be generally strong (Spearman’s rho = 0.65–0.99) [39, 42].

*The Metacognitions Questionnaire-30 (MCQ-30)* [43] is a shortened version of the original MCQ, consisting of 30 items designed to assess individual differences in metacognitive beliefs through self-report. The questionnaire includes five subscales: *cognitive confidence* (e.g., “I do not trust my memory”), *positive beliefs about worry* (e.g., “Worrying helps me to solve problems”), *cognitive self-consciousness* (e.g., “I monitor my thoughts”), *negative metacognitive beliefs about the uncontrollability and danger of worry* (e.g., “When I start worrying I cannot stop”), and *beliefs about the need to control thoughts* (e.g., “It is bad to think certain thoughts”). Each item is scored on a four-point Likert scale ranging from 1 (“I do not agree”) to 4 (“I totally agree”). Subscale scores range from 6 to 24, while total MCQ-30 scores range from 30 to 120, with higher scores reflecting greater levels of unhelpful metacognitions. Elevated MCQ-30 scores have been linked to increased depressive symptoms [44]. The MCQ-30 has demonstrated good internal consistency, with Cronbach’s alpha values between 0.72 and 0.93, and acceptable test-retest reliability, with Spearman’s rho values ranging from 0.59 to 0.87 [43]. In this study, Cronbach’s alpha values for the subscales ranged from 0.76 for *positive beliefs about worry* to 0.85 for *cognitive confidence*. For additional psychometric details, see Spada et al. [45].

### Data on demographic and stroke characteristics

The original study gathered stroke-related factors and medical information from participants’ medical records. Baseline data, including age, sex, and stroke subtype were retrieved from the National Norwegian Stroke Registry.

Information on education, marital status, degree of dependence in daily activities, and work status was obtained via questionnaires.

## Statistical analyses

The factor models were examined using Mplus version 8.9. Since the CFQ consists of 11 items covering two distinct aspects, i.e., physical and mental fatigue, we specified three conceptually different factor models. First, a conventional unidimensional model using a single factor to explain all CFQ item variances. Second, a correlated two-factor model with a physical and a mental fatigue factor accounting for their respective items. Third, a bi-factor model that can disentangle a general factor from the two specific factors. The factor model specifies all items to load on the general factor. In addition, the seven physical and four mental fatigue items additionally load on a specific physical and mental latent factor. These factors are estimated as orthogonal. A bi-factor approach examines if the fatigue symptoms are better accounted for by an overarching dimension plus two minor distinct dimensions rather than just a general dimension.

The fit of these models is assessed using chi-square statistics, the Root Mean Square Error of Approximation (RMSEA), Tucker Lewis Index (TLI), Bayesian Information Criterion, and Standardized Root Mean Square Residuals (SRMR). Lower values indicate better fit, except for TLI which is opposite.

For validation purposes, the latent factor scores of the best-fitting model were saved and imported into SPSS version 29 for further partial correlation and regression analyses.

Partial correlations: To examine the association between the CFQ sum scores and latent trait scores, all the psychosocial measured variables (i.e., another fatigue scale, anxiety, depression, stroke-specific quality of life, and five facets of meta-cognitive attitudes) and the stroke-related variables (i.e., previous stroke, dependency in daily activities, and stroke subtype), we conducted a partial correlation analyses that adjusted these relationships for socio-demographic differences (i.e., sex, age, education, work and cohabitation status. Correlations with *p*-values < 0.01 was interpreted.

Regression: A stepwise regression was conducted to simplify the interpretation of the correlational results by regressing the predictors and socio-demographic covariates on the observed CFQ sum score and saved latent trait scores. Given the high number of covariates relative to the sample size, the exploratory nature of this analysis and the high degree of collinearity between certain variables (e.g., anxiety and depression), a stepwise approach was preferred to minimize redundancy and reveal the most relevant covariates. This process was iterative by only including variables based on their statistical significance (*p* < 0.01). The standardized beta-weights and the adjusted R-Square (explained variance) are reported.

## Results

### Measurement models

Four measurement models were specified: (1) a single factor or unidimensional model with all 11 CFQ items loading onto a single latent factor (model 1), (2) a correlated two-factor model with the 7 physical and 4 mental items loading onto their respective two latent factors (model 2), and (3) a bi-factor model with all CFQ items loading onto a general factor, plus two specific factors for the 7 physical and 4 mental items loading onto their respective additional specific factors with all three factors being uncorrelated (model 3). (4) In addition, we specified a bi-factor model that omitted the specific factor for the 4 CFQ mental items (model 4) to examine their additional role in explaining more of the variance. According to Table 1, the conventional bi-factor (model 3) fit best across all fit indices. Model 4 also showed adequate fit due to low RMSEA and SRMR values and a marginally non-significant chi-square index (*p* = 0.06); hence, indicating that the specific mental factor may be discarded in a bi-factor setup if a simpler alternative is preferred.

**Table 1:** Model Fit of the measurement models (best fitting model in bold)

Model	χ^2^(df), *p*	BIC	RMSEA _90%CI_	TLI	SRMR
1: Single factor	125.5(44), < 0.001	2324.6	0.113 ._090 − 0.137_	0.862	0.071
2: Two factors	89.5(43), < 0.001	2283.5	0.087 ._061 − 0.112_	0.920	0.057
3: Bi-factor ^2^	29.2(33), **0.66**	**2261.1**	**0.0**_**0.0 − 0.051**_	**1.0**	**0.026**
4: Bi-factor ^1^	51.3(37), 0.06	2267.0	0.052 ._0 − 0.084_	0.971	0.039

Notes. χ^2^ = chi-square, df=degrees of freedom, *p* = *p*-value, BIC = Bayesian Information Criteria, RMSEA=Root Mean Square Error of Approximation, TLI=Tucker-Lewis Index, SRMR=Standardized Root Mean-square Residuals. ^2^ Bi-factor model with one general and two uncorrelated specific factors, ^1^ Bi-factor model with one general and one specific factor (specific physical factor retained, specific mental factor omitted)

### Factor loadings

Table 2 reports the factor loadings of the best-fitting model, that is: the bi-factor with one general (G-factor) and two specific factors (S-factors). The G-factor is most prominent given the magnitude of its factor loadings relative to the specific physical and mental fatigue loadings. The G-factor accounted well for the CFQ item score variances, except for the two mental items #9 (misspeaking) and #11 (memory), which had smaller general loadings and R-square values. These items are thus poorer indicators of general and mental fatigue and should be given less weight if interpreted individually. The specific physical factor accounted well for the CFQ items related to physical rest and restoration (e.g., rest, sleep, energy), but poorly for the physical capacity items (strength, weakness). The specific mental factor accounted well for item #10 (finding words) and somewhat for item #9 (misspeaking), but negligible for the two cognitive items (concentration and memory). By squaring these loadings, ~ 74% of the factor variance is specifically related to item #10 (finding words), which is relevant for the interpretation of this item. Of note, this item was also the strongest indicator of fatigue as the general and specific mental factors fully accounted for it (R-sq = 1.0). Since it also had one of the lowest intercept values as compared to the physical items, it is the most sensitive and discriminatory item indicative of general and mental fatigue. Taken together, the high general factor loadings and the superior fit of the bi-factor model support that fatigue can be validly represented through a general score. If further nuance is wanted, a latent specific physical score provides some extra information, whereas the latent specific mental factor score provides negligible extra information.

**Table 2:** Standardized bi-factor loadings

CFQ items	Latent intercept	G-factor	Physical factor	Mental factor	*R*-sq
1 – Feeling tired	1.54	0.75	0.46		0.77
2 – Needs rest	1.57	0.60	0.72		0.87
3 – Feeling sleepy	1.46	0.61	0.57		0.69
4 – Getting started	1.45	0.71	0.30		0.59
5 – Lacking energy	1.55	0.78	0.41		0.78
6 – Muscle strength	1.46	0.72	0.19		0.56
7 – Feeling weak	1.40	0.83	0.07		0.69
8 – Concentration	1.31	0.75		0.14	0.58
9 – Misspeaking	1.08	0.35		0.37	0.26
10 – Finding words	1.40	0.62		0.78	1.00
11 – Memory	1.37	0.54		0.23	0.34
Loading: M (SD)		0.66 (0.12)	0.39 (0.19)	0.38 (0.22)	

Notes. Latent intercept = the expected item mean score when all three latent factor scores equal zero, which represents the patients’ average level of fatigue. M=mean, SD=standard deviation

### Correlations between CFQ sum and factor scores, and demographic variables

Simple correlation analyses (Table 3) show a strong overlap between the G-factor and the CFQ overall sum score (*r* = 0.96), the CFQ physical sum score (*r* = 0.92) and the CFQ mental sum score (*r* = 0.81). The use of separate sum scores for the physical and the mental CFQ items is thus less supported as they all correlate strongly with the G-factor. The estimated specific factor scores instead contribute more uniquely to the fatigue construct as their correlation with their respective CFA sum score equivalents are markedly lower (*r* = 0.53 and *r* = 0.53), as are their respective correlations with the overall CFQ sum score (*r* = 0.40 and *r* = 0.17). This means that the specific factor scores measure aspects of fatigue that is different from their sum scores.

Most of the demographic variables were not significantly related to the CFQ variables, except for gender (men reporting less fatigue). Gender showed a comparable correlational pattern with the total and physical sum scores as with the G- and the physical factor scores (Table 3).

**Table 3:** Correlation coefficients between CFQ sum and latent factor scores

	Sum total	Sum physical	Sum mental	G-factor	Specific physical	Specific mental
CFQ variables						
Sum physical	**0.97**					
Sum mental	**0.81**	**0.65**				
G-factor	**0.96**	**0.92**	**0.81**			
Physical factor	**0.40**	**0.53**	− 0.01	0.17		
Mental factor	0.17	0.00	**0.53**	0.11	− 0.13	
Demographics						
Men (vs. women)	**− 0.29**	**− 0.32**	− 0.13	**− 0.22**	**− 0.35**	0.03
Age 56+ (vs. 18–55 yrs)	− 0.17	− 0.16	− 0.16	− 0.16	− 0.11	0.00
Age 75+ (vs. 18–55 yrs)	0.02	0.04	− 0.04	0.03	− 0.02	− 0.13
Previous stroke (vs. not)	0.07	0.06	0.07	0.09	− 0.07	0.08
Educ upper (vs. lower)	− 0.01	0.01	− 0.06	− 0.04	0.13	− 0.01
Educ univ (vs. lower)	− 0.03	− 0.06	0.04	0.00	− 0.14	0.09
Work (vs. retired/sick)	0.01	− 0.03	0.09	0.01	0.01	0.07
Living alone (vs. cohabit)	0.09	0.12	− 0.01	0.09	0.08	− 0.18

Notes. Coefficients in bold = *p* < 0.01

### Validation analyses

Partial correlation analyses: The correlational patterns in Table 4 show strong congruence between the total CFQ sum score and the G-factor score columns. The congruence was markedly weaker for the physical sum and the specific physical factor score, but the directions were similar, except for the MCQ cognitive confidence measure, which showed the opposite direction. There was no congruence between the mental sum, and the specific mental factor score except for the MCQ cognitive confidence measure. These findings indicate that a simple sum score of the CFQ items represents a general fatigue factor well, whereas the creation of separate raw sum scores for the physical and, in particular, the mental CFQ items provides mediocre to poor additional nuance to fatigue. The high congruence in correlation coefficients for the physical and the total sum scores indicates the same.

**Table 4:** Partial correlation analyses of the CFQ variables with the criterion-related variables

Criterion-related	Sum total	Sum physical	Sum mental	G-factor	Specific physical	Specific mental
Haemorrhagic stroke	0.03	0.02	0.05	0.01	0.04	*0.21*
modified Rankin Scale	**0.47**	**0.49**	**0.30**	**0.46**	0.20	0.10
FSS	**0.51**	**0.55**	**0.30**	**0.47**	**0.32**	− 0.08
HADS anxiety	**0.47**	**0.45**	**0.40**	**0.44**	0.20	0.14
HADS depression	**0.61**	**0.63**	**0.40**	**0.57**	**0.29**	0.03
SS-QOL PH	**− 0.46**	**− 0.49**	**− 0.29**	**− 0.48**	− 0.15	− 0.02
SS-QOL CSM	**− 0.62**	**− 0.63**	**− 0.45**	**− 0.61**	− 0.21	− 0.08
MCQ pos beliefs	0.08	0.07	0.09	0.07	0.02	0.07
MCQ neg beliefs	**0.26**	**0.25**	0.21	**0.24**	0.15	0.00
MCQ cogn confidence	**0.32**	0.21	**0.49**	**0.32**	− 0.15	**0.29**
MCQ need to control	0.12	0.13	0.07	0.12	0.03	− 0.07
MCQ cogn self-conscious	0.01	0.04	− 0.06	0.00	0.09	− 0.08

Notes. These coefficients are adjusted for demographics. Coefficients in bold = *p* < 0.01

FSS = Fatigue Severity Scale, HADS = Hospital Anxiety and Depression Scale, SS-QOL = Stroke-Specific Quality of Life scale, SS-QOL PH = Physical Health component, SS-QOL CSM = Cognitive-Social-Mental component MCQ = Meta-Cognitive Questionnaire, MCQ pos = positive beliefs about worry, MCQ neg = negative beliefs about worry, MCQ cc = cognitive confidence, MCQ nc = need to control thoughts, MCQ csc = cognitive self-consciousness

Stepwise regression analyses: A finding that would strengthen the utility of estimating separate general and specific physical and mental factor scores, would be a different prediction of these scores compared to their sum score equivalents. When successively regressing the demographic and criterion-related variables on the six CFQ scores (Table 5), the same predictors explained the overall sum score as the general factor score (G-factor).

For the physical sum score, the Stroke-Specific Quality of Life (SS-QOL) scale and HADS depression were significant predictors, whereas these covariates did not contribute to the specific physical factor score. In addition, sex (more women) contributed significantly to the factor but not the sum score. The physical sum score can thus be interpreted as overlapping with the overall sum score, as concluded above, with only a minor contribution from the FSS. The physical factor score offers a more distinct measure of physical fatigue, as it is not influenced by depression or the cognitive/social/mental facets of the SS-QOL, but it is affected by sex, with women reporting higher scores. For the CFQ mental scores, HADS anxiety contributed to the sum, but not the factor score. Again, the specific mental factor score is a more specific indication of mental fatigue by excluding anxious mood.

**Table 5:** Demographic and criterion-related variables regressed on the total sum score, G-Factor, and the specific physical and mental fatigue factor scores

Predictors	Sum total	Sum physical	Sum mental	G-factor	Specific physical	Specific mental
Adj R-square	51.6%	57.3%	33.8%	46.4%	22.4%	8.0%
SS-QOL CSM	− 0.40***	− 0.30***		− 0.40***		
HADS depression	0.40***	0.37***		0.36***		
FSS		0.22**			0.33***	
Men (vs. women)					− 0.26**	
MCQ cogn confidence			0.38***			0.30***
HADS anxiety			0.35***			

Notes. These coefficients are adjusted for demographics. Coefficients in bold = *p* < 0.01. MCQ

## Discussion

The psychometric analyses of the CFQ yielded superior fit for a *bi-factor* model describing a *general* fatigue factor (i.e., G-factor) while simultaneously accounting for two distinct factors of physical and mental fatigue. This model outperformed the more commonly reported unidimensional and the two-factor correlated models in terms of model fit [12, 18]. This implies that the CFQ items are good indicators of general fatigue, except for two CFQ items (i.e., misspeaking; “Do you make slips of the tongue when speaking?” and “How is your memory?”) belonging to the mental fatigue dimension, which were less well accounted for – possibly also needing revision. The specific physical factor explained a noteworthy degree of additional item variance, whereas the contribution by the specific mental factor was minor. Since the simple sum scores of all CFQ items, the seven physical or the four mental fatigue items all correlated strongly with the latent G-factor trait score, the creation of separate sum scores for physical and mental fatigue stands out as redundant. If a fatigue assessment requires separating its characteristics into general, physical, and mental aspects, a bi-factor derivation is mandatory. The results show that a bi-factor estimation provides a more nuanced fatigue assessment than a simple subscale sum scores is capable of, but the extra contribution is limited. The CFQ items primarily reflect a general fatigue factor, suggesting that the scale can be essentially treated as unidimensional.

Furthermore, we found that the most sensitive and discriminatory CFQ item was the mental fatigue item “Do you find it more difficult to find the right word?”, which also may indicate that the mental fatigue dimension represents problems with word retrieval or verbal fluency, alongside misspeaking, concentration and memory symptoms. In a stroke population, this specific item may be problematic as 46% of participants four years after stroke in the present study reported “slight problems” or “some problems” on a comparable item from the SS-QOL “Did you have trouble finding the words you wanted to say?”. Accordingly, this CFQ item may not be an ideal indicator of fatigue in the stroke population, as difficulties in word retrieval could stem from stroke-related language deficits rather than fatigue. Another consideration is that the updated version of CFQ is the only version translated into Norwegian. As mentioned in the methods section, the authors of the CFQ state that both item versions can be used without altering the scale [25]. Consequently, future validation for the stroke population should consider the original CFQ version with the item “Do you have problems thinking clearly?” instead of the item “Do you find it more difficult to find the right word?”. This underscores the need for considering stroke-specific items that better differentiate between cognitive and language deficits due to neurological damage and those due to fatigue.

Although the CFQ may detect and differentiate between physical and mental fatigue in some populations, this may be more challenging in a stroke population. As discussed by Kjeverud et al. [13], the four items intended to measure mental fatigue might reflect subjective cognitive deficits more than mental fatigue. For example, problems thinking clearly and memory problems might as well be cognitive shortfalls as fatigue. The item “do you have problems starting things?” is part of measuring physical fatigue, but this question may also represent cognitive deficits. Furthermore, the item concerning muscle weakness in the physical fatigue subscale may reflect post-stroke physical consequences, i.e., paresis, rather than fatigue. The prevalence of post-stroke fatigue (PSF) has been reported to affect approximately 48% of stroke survivors [9], and the development of stroke-specific measures of fatigue might be necessary to accomplish more specific measures of fatigue for this population. Furthermore, future studies should examine whether supplementary or alternative mental fatigue items provide a more accurate assessment of mental fatigue after stroke.

While the CFQ can be practiced as unidimensional, the bifactor separation may still offer added value in certain clinical or assessment contexts. Since the latent specific factors are “pure” indicators of physical and mental fatigue, with the effect of the general fatigue removed, they may indicate which of the two specific factors are most affected in a patient beyond the effect of the general fatigue level. One example is the additional burden of depression, which in the present analysis was strongly correlated with the latent general fatigue score (*r* = 0.57), moderately with the latent physical score (*r* = 0.29), but not correlated with the latent mental score (*r* = 0.03), which indicates that consideration of mental fatigue is irrelevant after accounting for the level of general fatigue when considering the implications of possible comorbid depression. This distinction is entirely missed when using simple sum scores for assessing general, physical, and mental fatigue. Despite the minor contributions of the latent specific factor scores, these scores may be more useful in assessing fatigue among patients with more complex or comorbid symptom constellations.

In intervention studies, a bifactor separation may help clarify the extent to which favorable changes in latent specific factors occur in addition to the general fatigue level. This distinction may be relevant in rehabilitation of stroke populations offering both physical and cognitive rehabilitation, but with the interest in understanding which of the three latent factors such interventions benefit most from.

### Study strengths and limitations

To our knowledge, the current study is the first to explore the factor structure of the CFQ in a stroke population. Such studies help determine whether the scale’s dimensions align with those identified in other populations or if unique factors emerge in the context of PSF. Enhancing our understanding of core PSF instruments and their relationship to other characteristics is crucial for advancing clinical practice and PSF research. This knowledge has significant implications for improving measurement approaches, refining theoretical models, and developing effective treatments.

A potential limitation of the present study is the risk of non-response bias, as individuals with more severe strokes were more likely to not participate in the parent study from which this study recruited its sample. Consequently, the findings are most applicable to individuals with mild to moderate strokes, while their generalizability to those with more severe strokes may be more limited. Another limitation is the relatively small and homogenous Norwegian sample, which also limits generalizability. Patient-Reported Outcome Measures (PROMs) are valuable for capturing the patient’s perspective on their health and well-being; however, they have limitations. PROMs require a certain level of reading comprehension, which may disadvantage individuals with stroke-related visual, cognitive and- /or language impairments. Limitations in comprehension could lead to misinterpretation of questionnaire items. The current study only assesses PSF at a single time point, four years after stroke. Since fatigue may evolve over time, assessments at multiple time points, including earlier ones, would provide a more complete understanding. Nevertheless, few studies have assessed long-term PSF, and this study adds valuable knowledge of long-term PSF-assessment.

## Conclusion

The findings in this study indicate that a simple overall fatigue score aligns well with the estimated general factor score (the G factor), as does the physical sum score. We conclude that creating separate physical and mental CFQ sum scores does not add information beyond the overall score, whereas estimation of physical and mental factor scores provides a more nuanced assessment. However, given the limited clinical magnitude of these contributions, the CFQ may be justifiably regarded as sufficiently unidimensional for clinical assessment.

This implies that clinicians and researchers can use the CFQ total score as a robust measure of PSF without losing essential information. Further studies may be warranted to explore whether item modifications or alternative models could provide a more reliable and valid assessment of mental fatigue. Future research may also benefit from including larger, more diverse samples to improve generalizability.

## Data Availability

The dataset analysed during the current study are not publicly available due to ethical restrictions and personal data protection. Data are available from the Data Protection Officer (contact personvernombudet@unn.no) for researchers who meet the criteria for access to confidential data.

## Abbreviations

PSF: Post stroke fatigue
mRS: Modified rankin scale
FSS: Fatigue severity scale
HADS: Hospital anxiety and depression scale
SS-QOL: Stroke–specific quality of life scale
MCQ-30: Metacognitions questionnaire–30
CFQ: Chalder fatigue questionnaire
RMSEA: Root mean square error of approximation
TLI: Tucker lewis index
SRMR: Standardized root mean square residual
SPSS: Statistical package for the social sciences
ICD-10: International classification of diseases, 10th Edition
Cronbach's alpha: Measure of internal consistency/reliability
Spearman’s rho: A measure of correlation (non–parametric)
BIC: Bayesian information criterion
SD: Standard deviation
p: p–value (statistical significance)
SS-QOL PH: Physical health component
SS-QOL CSM: Cognitive–social–mental component
MCQ: Metacognitions questionnaire
MCQ pos: Positive beliefs about worry
MCQ neg: Negative beliefs about worry
MCQ cogn confidence: Cognitive confidence
MCQ need to control: Need to control thoughts
MCQ cogn self-conscious: Cognitive self–consciousness
R-sq: R–squared
Adj R-square: Adjusted r–squared
PROMs: Patient–reported outcome measures
CFA: Confirmatory factor analysis
EFA: Exploratory factor analysis
